# Risk factors for hepatitis C seropositivity among young people who inject drugs in New York City: Implications for prevention

**DOI:** 10.1371/journal.pone.0177341

**Published:** 2017-05-19

**Authors:** Benjamin Eckhardt, Emily R. Winkelstein, Marla A. Shu, Michael R. Carden, Courtney McKnight, Don C. Des Jarlais, Marshall J. Glesby, Kristen Marks, Brian R. Edlin

**Affiliations:** 1 Division of Infectious Diseases, Department of Medicine, Weill Cornell Medicine, New York, New York, United States of America; 2 Institute for Infectious Disease Research, National Development and Research Institutes, New York, New York, United States of America; 3 Department of Psychiatry, Icahn School of Medicine at Mount Sinai, New York, New York, United States of America; 4 Baron Edmond de Rothschild Chemical Dependency Institute, Department of Psychiatry, Icahn School of Medicine at Mount Sinai, New York, New York, United States of America; 5 Department of Medicine, Weill Cornell Medicine, New York, New York, United States of America; University of Cyprus, CYPRUS

## Abstract

**Background:**

Hepatitis C virus (HCV) infection remains a significant problem in the United States, with people who inject drugs (PWID) disproportionately afflicted. Over the last decade rates of heroin use have more than doubled, with young persons (18–25 years) demonstrating the largest increase.

**Methods:**

We conducted a cross-sectional study in New York City from 2005 to 2012 among young people who injected illicit drugs, and were age 18 to 35 or had injected drugs for ≤5 years, to examine potentially modifiable factors associated with HCV among young adults who began injecting during the era of syringe services.

**Results:**

Among 714 participants, the median age was 24 years; the median duration of drug injection was 5 years; 31% were women; 75% identified as white; 69% reported being homeless; and 48% [95% CI 44–52] had HCV antibodies. Factors associated with HCV included older age (adjusted odds ratio [AOR], 1.99 [1.52–2.63]; *p*<0.001), longer duration of injection drug use (AOR, 1.68 [1.39–2.02]; *p*<0.001),more frequent injection (AOR, 1.26 [1.09–1.45]; *p* = 0.001), using a used syringe with more individuals (AOR, 1.26 [1.10–1.46]; *p* = 0.001), less confidence in remaining uninfected (AOR, 1.32 [1.07–1.63]; *p*<0.001), injecting primarily in public or outdoors spaces (AOR, 1.90 [1.33–2.72]; *p*<0.001), and arrest for carrying syringes (AOR, 3.17 [1.95–5.17]; *p*<0.001).

**Conclusions:**

Despite the availability of harm reduction services, the seroprevalence of HCV in young PWID in New York City remained high and constant during 2005–2012. Age and several injection behaviors conferred independent risk. Individuals were somewhat aware of their own risk. Public and outdoor injection and arrest for possession of a syringe are risk factors for HCV that can be modified through structural interventions.

## Introduction

Hepatitis C virus (HCV) is the most common blood-borne pathogen in the United States, with chronic infection being the leading cause of cirrhosis and liver cancer[1]. In 2012, deaths associated with HCV infection surpassed all 60 other nationally notifiable infectious disease deaths combined[2]. Efficiently transmitted via contaminated needles and syringes, HCV is endemic in people who inject drugs (PWID). PWID who share needles, syringes, or other injection equipment are at the highest risk for contracting and transmitting HCV with incidence rates as high as 40 per 100 person-years [1,3]. HCV prevalence in injection drug using populations varies widely around the world with an estimated 70–90% of PWID in the US infected[4–9]. Needle and syringe programs developed to reduce the transmission of blood-borne infections such as HIV, hepatitis B, and hepatitis C have been instituted in 33 states, operating within 196 cities in the United States. Data suggest needle and syringe programs have reduced the rates of HCV transmission in PWID where services are widely available. Incidence rates decreased 15–43%[3,10,11], although rates still remain as high as 10–25 infections per 100 person-years[12–15].

The United States has been experiencing a dramatic opioid epidemic for more than a decade, affecting young people especially, spurring an alarming increase in HCV transmission as opioid-dependent people turn to injection. This has widely affected communities with little or no access to HIV prevention interventions, resulting in rapid spread of HCV[16], and putting them at risk for HIV transmission, as demonstrated by the recent HIV outbreak in Indiana[17]. Between 2002 and 2013 heroin use in the United States has increased by 63%, with the largest increase (109%) in individuals age 18–25 [18]. This rise in heroin use in young people has been accompanied by increases in HCV infection. Reported cases of acute HCV increased more than 2.5 times from 2010–2014, with increases greatest among people age 20–29[19]. Although these increases have been proportionately greater in suburban and rural areas, 67% of reported acute hepatitis C cases in young people in the United States during 2006–2012 were in urban counties[20].

Certain demographic characteristics and risk behaviors have consistently been associated with HCV infection in PWID, including older age, longer duration and greater frequency of injection drug use, and injection of cocaine[21–25]. However little is known about why HCV continues to spread among people who use drugs where there is access to existing prevention strategies. This information will be particularly important as efforts are made to reduce the spread of HCV among the many communities newly affected by the opioid epidemic.

We conducted a study to examine the reasons for continued HCV transmission amongst young people who inject drugs in a location where needle exchange was already available. In New York City, 14 syringe services programs provide access to sterile injection equipment, education, testing for HIV and HCV, and other services at 51 sites across the city[26]. These serve an estimated 106,849 injection drug users, 21% of whom are under the age of 29 [27]. In addition, New York changed its laws to permit drug users to purchase sterile injecting equipment at pharmacies and many PWID, particularly younger ones, do obtain needles and syringes at pharmacies. This study explored mechanisms of HCV spread and potentially modifiable risk factors in a group of young people injecting drugs in New York City.

## Materials and methods

Between 2005 and 2012, the Swan Project recruited young people who inject drugs (PWID) on the Lower East Side of Manhattan. The Lower East Side has long been home to a community of young, often homeless, PWIDs and is served by two syringe exchange programs. Participants were recruited through street outreach referral from community-based agencies such as syringe exchange programs, and participant word-of-mouth. During the early years of the study, outreach workers approached potential participants on the streets and in parks, told them about the study, screened them for eligibility, and referred those who were interested and eligible to the study. Eligible participants were between 18 to 35 years of age or had injected drugs for ≤5 years, and had injected illicit drugs in the 30 days before enrollment. Participants were interviewed, tested for HCV, and screened for a prospective cohort study on the acquisition of new HCV infection [28]. The primary objective of the Swan Study was to follow HCV-negative PWID prospectively to evaluate risk factors for incident infection. This study reports cross-sectional data collected at baseline on all participants with HCV antibody data available.

At enrollment, all participants underwent a face-to-face interview using a standardized questionnaire and were tested for HCV antibody. Blood was collected from each participant and tested for HCV antibodies by second (HCV EIA 2.0, Abbott Laboratories, Abbott Park, IL) or third (HCV 3.0 ELISA and RIBA HCV 3.0, Ortho Clinical Diagnostics, Raritan, NJ) generation tests, and for HIV antibodies for consenting participants. Written consent was obtained from all subjects. The protocol was approved by the Institutional Review Boards of Weill Cornell Medical College, Beth Israel Medical Center, and SUNY Downstate College of Medicine.

Participant characteristics were examined to identify risk factors for HCV seropositivity. Chi-square testing was used to examine categorical variables, and the Mantel-Haenszel chi-square test for linear trend was used for ordinal variables. Multivariable logistic regression analysis was performed to identify factors independently associated with HCV seropositivity. Collinearity of variables was assessed with the Pearson correlation coefficient; where the coefficient was >0.5, only one of the collinear variables was included in the model. Continuous variables with skewed distribution were log transformed. A hierarchical approach was used to select variables for inclusion in multiple logistic regression models. Factors representing potential mechanisms of transmission (explanatory variables) were first assessed, and a single model was fitted containing only effects with p≤0.1. Next, social, behavioral, and contextual factors were added to the model individually (in separate models) to assess the contribution of each one that was independent of the explanatory injection practices (but not necessarily of each other). All analyses were performed using STATA software (v 13.1; StataCorp, College Station, TX).

## Results

The Swan Project recruited 731 participants who met the eligibility criteria. Blood could not be obtained from 10 and HCV antibody test results were inconclusive for 7, leaving 714 who had HCV antibody results available and were included in the analysis. Of the 714 participants, the median age was 24 years (mean 24.9), with 13 (2%) older than 35-years-old. Three-quarters of the participants identified as white, two-thirds were men, and nearly three-quarters had a high school education or the equivalent (Table 1). Two-thirds reported being homeless. The median number of injections during the prior 30 days was 60 (mean 87.8). HIV antibodies were present in only 4 (0.68%) of the 584 participants with available HIV antibody data.

**Table 1:** HCV seroprevalence by demographics characteristics, young people who inject drugs, New York City 2005–2012.

Variable	No. (%) of participants	No. (%) HCV Ab (+)	Unadjusted OR	95% CI		p
**TOTAL**	714 (100%)	343 (48.0%)				
Age, y						<0.001*
18–19	99 (13.9%)	21 (21.2%)	1.00	REF		
20–24	279 (39.1%)	116 (41.6%)	2.64	1.54	4.52	
25–29	207 (29.0%)	125 (60.4%)	5.66	3.24	9.88	
30–34	103 (14.4%)	66 (64.1%)	6.63	3.54	12.41	
≥35 [35–55]	26 (3.6%)	15 (57.7%)	5.06	2.03	12.65	
Gender						0.209
Male	486 (68.1%)	241 (49.6%)	1.00	REF		
Female	222 (31.1%)	99 (44.6%)	0.82	0.59	1.13	
Ethnicity						0.137
White	532 (74.6%)	265 (49.8%)	1.00	REF		
Black	25 (3.5%)	7 (28.0%)	0.39	0.16	0.95	
Latino	108 (15.2%)	108 (48.2%)	0.94	0.62	1.42	
Asian	7 (1.0%)	1 (14.3%)	0.17	0.02	1.4	
Mixed	31 (4.4%)	13 (41.9%)	0.73	0.35	1.52	
Other	10 (1.4%)	5 (50.0%)	1.01	0.29	3.52	
Currently homeless						0.006
No	223 (31.2%)	90 (40.3%)	1.00	REF		
Yes	491 (68.8%)	253 (51.5%)	1.57	1.14	2.17	
High School Diploma or GED						0.148
No	197 (29.6%)	86 (43.7%)	1.00	REF		
Yes	517 (72.4%)	257 (49.7%)	1.28	0.92	1.77	
Currently employed						0.031
No	677 (95.0%)	332 (49.0%)	1.00	REF		
Yes	36 (5.0%)	11 (30.6%)	0.46	0.22	0.94	

Ab = antibody, OR = odds ratio, CI = confidence interval

* Mantel-Haenszel chi-square test for trend

Of the 714 participants, 343 (48.0%, 95% CI 44.4–51.7) had a positive HCV antibody test, indicating either past or present HCV infection; 163 (47.5%) of these, based on self-report, were new diagnoses. There was no association with season or date of enrollment (data not shown). Seroprevalence increased markedly with increasing age. Social factors significantly associated with HCV antibodies included being homeless and being unemployed.

**Fig 1:**
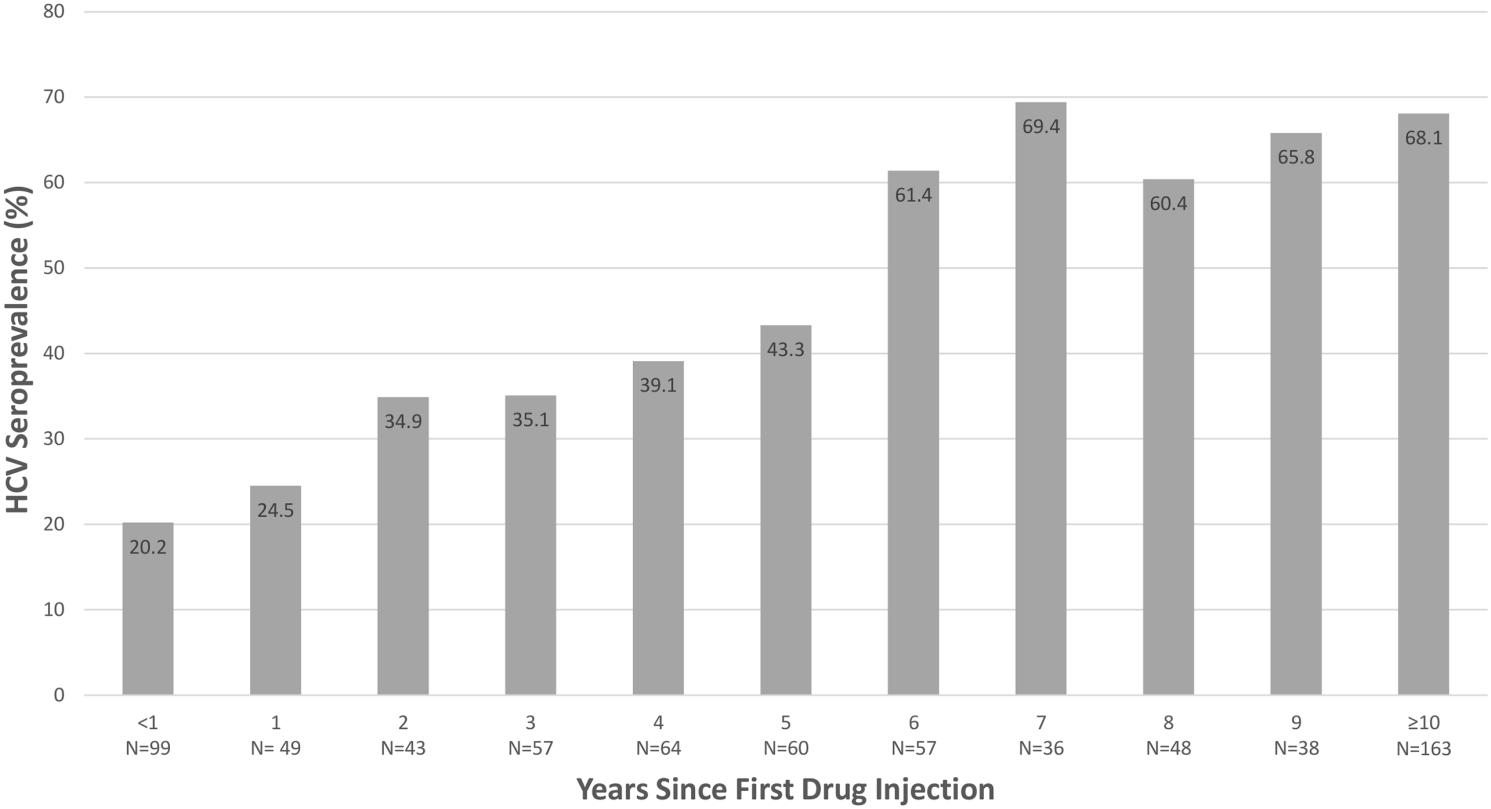
HCV seroprevalence by duration of injection drug use, New York City, 2005–2012. Seroprevalence didn’t rise after 6–7 years, suggesting that people who didn’t get infected during their early years of injection were much less likely to do so thereafter.

Study participants had been injecting drugs for a median of 5 years (mean 6.0). Those who had injected longer were at increased risk for having HCV antibodies (Table 2)(Fig 1). Most (80.4%) participants had been given their first injection by another person. If this person was ≥30 years old, the participant was more likely to have been infected. Only 67.5% of participants knew that HCV could be transmitted by sharing needles when they began injecting, and those who did not were at increased risk. Participants were at least somewhat able to estimate the magnitude of their own risk; excluding those who reported a prior positive HCV test, participants’ confidence in their ability to avoid infection was associated with a negative HCV antibody test.

**Table 2:** HCV seroprevalence by injection drug use characteristics, young people who inject drugs, New York City 2005–2012.

Variable	No. (%) of participants	HCV Ab (+)	Unadjusted OR	95% CI		p
**TOTAL**	714 (100%)	343 (48.0%)				
Years since first drug injection (years)						<0.001*
<1	99 (13.9%)	20 (20.2%)	1.00	REF		
1–4	213 (29.8%)	72 (33.8%)	2.02	1.14	3.56	
5–9	239 (33.5%)	140 (58.6%)	5.59	3.21	9.72	
≥10	163 (22.8%)	111 (68.1%)	8.43	4.67	15.22	
Person who administered first injection						0.58
Self	140 (19.6%)	65 (46.4%)	1.00	REF		
Primary sex partner	96 (13.4%)	48 (50.0%)	1.15	0.69	1.94	
Other sex partner	14 (2.0)	7 (50.0%)	1.15	0.38	3.46	
A relative or close friend	327 (45.8%)	150 (43.7%)	0.98	0.66	1.45	
Dealer, gallery operator, hit doctor	15 (2.1%)	8 (53.3%)	1.32	0.45	3.83	
Acquaintance	116 (16.2%)	60 (51.7%)	1.24	0.76	2.02	
Other	6 (0.8%)	5 (83.3%)	5.77	0.66	50.66	
Age of person who administered first injection (years)						0.039*†
*Self*	140 (19.6%)	65 (46.4%)	-	-		
<20	194 (27.2%)	87 (44.9%)	1.00	REF		
20–24	174 (24.4%)	78 (44.8%)	1.00	0.66	1.51	
25–29	101 (14.1%)	50 (49.5%)	1.21	0.74	1.95	
≥30	96 (13.4%)	58 (60.4%)	1.88	1.14	3.09	
Before first injection knew HIV could be transmitted by sharing needles						0.2
Did not know	57 (8.0%)	32 (56.1%)	1.00	REF		
Knew	657 (92.0%)	311 (47.3%)	0.70	0.41	1.21	
Before first injection knew hepatitis could be transmitted by sharing needles						<0.001
Did not know	232 (32.5%)	140 (60.3%)	1.00	REF		
Knew	482 (67.5%)	203 (42.1%)	0.48	0.35	0.66	
Before first injection knew hepatitis could be transmitted by sharing cottons, cookers, or rinse water						0.1
Did not know	458 (64.3%)	230 (50.2%)	1.00	REF		
Knew	254 (35.7%)	111 (43.7%)	0.77	0.57	1.05	
Confidence in avoiding hepatitis C virus infection						<0.001‡
Extremely confident	180 (32.4%)	45 (25.0%)	1.00	REF		
Somewhat confident	230 (41.4%)	70 (30.4%)	1.31	0.85	2.04	
A little confident	72 (13.0%)	29 (40.3%)	2.02	1.13	3.61	
Not confident at all	74 (13.3%)	49 (66.2%)	5.88	3.27	10.59	
*Self-reported HCV-positive*	180	165 (91.7%)	-	-	-	

Ab = antibody, OR = odds ratio, CI = confidence interval

*Mantel-Haenszel chi-square test for trend;

^†^Excludes those who self-injected;

^‡^Excludes self-reported positive

The 57.4% of our participants who reported having overdosed were 2.69 [95% CI: 1.98–3.66] times more likely to have HCV antibodies than those who had never overdosed (Table 3). The 19% of our participants who had been arrested solely for drug residue or possession of a needle or syringe were nearly five times [OR 4.77, 95% CI: 3.09–7.36] more likely than the others to have HCV antibodies.

**Table 3:** HCV seroprevalence by injection practices and other experiences, young people who inject drugs, New York City, 2005–2012.

Variable	No (%) of participants	No (%) HCV Ab (+)	Unadjusted OR	95% CI		p
**TOTAL**	714 (100%)	343 (48.0%)				
Ever injected heroin						0.5
No	6 (0.8%)	2 (33.3%)	1.00	-		
Yes	708 (99.2%)	341 (48.2%)	1.85	0.34	10.21	
Ever injected cocaine or crack						<0.001
No	143 (20.0%)	34 (23.8%)	1.00	-		
Yes	571 (80.0%)	309 (54.1%)	3.78	2.49	5.75	
Ever injected pharmaceutical pain killers						0.009
No	616 (86.3%)	284 (46.1%)	1.00	-		
Yes	98 (13.7%)	59 (60.2%)	1.77	1.15	2.73	
Ever injected crystal meth						0.001
No	429 (60.1%)	185 (43.1%)	1.00	-		
Yes	285 (39.9%)	158 (55.4%)	1.64	1.21	2.22	
Ever injected ketamine						0.047
No	510 (71.4%)	233 (45.7%)	1.00	-		
Yes	204 (28.6%)	110 (53.9%)	1.39	1	1.93	
Ever overdosed						<0.001
No	304 (42.6%)	104 (34.2%)	1.00	-		
Yes	410 (57.4%)	239 (58.3%)	2.69	1.98	3.66	
Ever been given money or drugs in exchange for sex						0.02
No	512 (71.7%)	232 (45.3%)	1.00	-		
Yes	202 (28.3%)	111 (55.0%)	1.47	1.06	2.04	
Ever arrested solely for drug residue or possession of syringe or needle						<0.001
No	579 (81.1%)	239 (41.3%)	1.00	-		
Yes	135 (18.9%)	104 (77.0%)	4.77	3.09	7.36	

Injection practices during the past 6 months that were associated with increased HCV antibody positivity included higher injection frequency, more frequent use of needle/syringes previously used by another person, and dividing drug with syringes previously used by other people (Table 4). 53.9% of our study participants injected most frequently in public or outdoor locations, and this practice was associated with HCV antibody positivity. Drawing drugs from a previously used cooker, cotton, or rinse water and splitting drugs with a previously used syringe were all associated with HCV seropositivity.

**Table 4:** HCV seroprevalence by recent injection practices, young people who inject drugs, New York City, 2005–2012.

Variable	No (%) of participants	HCV Ab (+)	Unadjusted OR	95% CI		p
**TOTAL**	714 (100%)	343 (48.0%)				
Injection frequency (injections/month) in past 30 days						<0.001*
<30	219 (30.7%)	86 (39.3%)	1.00	-		
30–59	111 (15.6%)	43 (38.7%)	0.98	0.61	1.56	
60–89	110 (15.4%)	56 (50.9%)	1.60	1.01	2.55	
90–149	148 (20.7%)	85 (57.4%)	2.09	1.37	3.19	
150–299	96 (13.5%)	52 (54.2%)	1.83	1.13	2.97	
≥300	30 (4.2%)	21 (70.0%)	3.61	1.58	8.28	
Location where injected drugs most (past 6 months)						0.01
Your or primary partner's home	187 (26.2%)	72 (38.5%)	1.00			
Home of a friend or relative	79 (11.1%)	32 (40.5%)	1.09	0.64	1.86	
Public or outdoor space	358 (53.9%)	206 (53.5%)	1.84	1.29	2.62	
Other indoor space	37 (5.2%)	19 (51.4%)	1.68	0.83	3.42	
Other	26 (3.6%)	14 (53.9%)	1.86	0.82	4.25	
No. times injected with needle/syringe used previously by someone else(past 6 months)						<0.001*
Never	325 (45.6%)	131 (40.3%)	1.00	-		
1–3 times	134 (18.8%)	59 (44.0%)	1.16	0.78	1.75	
4–9 times	80 (11.2%)	41 (51.3%)	1.56	0.95	2.54	
10–25 times	69 (9.7%)	47 (68.1%)	3.16	1.82	5.50	
>25 times	105 (14.7%)	65 (61.9%)	2.41	1.53	3.78	
No. people who used a needle/syringe before participant (past 6 months)						<0.001*
None	333 (48.2%)	136 (40.8%)	1.00	-		
1 person	168 (24.3%)	85 (50.6%)	1.48	1.02	2.15	
2 people	59 (8.5%)	26 (44.1%)	1.14	0.65	1.99	
3 people	43 (6.2%)	28 (65.1%)	2.7	1.39	5.25	
4–9 people	65 (9.4%)	41 (63.1%)	2.47	1.43	4.29	
10–25 people	18 (2.6%)	14 (77.8%)	5.07	1.63	15.73	
>25 people	5 (0.7%)	4 (80.0%)	5.79	0.64	52.41	
No. times divided drugs by drawing into syringe used on a previous occasion by someone else (past 6 months)						<0.001*
Never	503 (70.5%)	214 (42.5%)	1.00	-		
1–3 times	66 (9.2%)	30 (45.5%)	1.13	0.67	1.88	
4–9 times	41 (5.7%)	27 (65.9%)	2.60	1.33	5.09	
10–25 times	49 (6.9%)	31 (63.3%)	2.33	1.27	4.27	
>25 times	55 (7.7%)	41 (74.6%)	3.95	2.10	7.44	
No. people divided drugs with by drawing into syringe used by someone else before participant (past 6 months)						<0.001*
None	485 (70.9%)	206 (42.5%)	1.00	-		
1 person	135 (19.7%)	71 (52.6%)	1.50	1.02	2.20	
2 people	30 (4.4%)	22 (73.3%)	3.72	1.63	8.53	
3 people	11 (1.6%)	8 (72.7%)	3.61	0.95	13.78	
4–9 people	18 (2.6%)	15 (83.3%)	6.77	1.93	23.70	
10–25 people	3 (0.4%)	3 (100%)	-	-	-	
>25 people	2 (0.3%)	2 (100%)	-	-	-	
No. times drew from drug solution in cooker accessed previously by someone else (past 6 months)						<0.001*
Never	278 (38.9%)	124 (44.6%)	1.00	-		
1–3 times	132 (18.5%)	49 (37.1%)	0.73	0.48	1.12	
4–9 times	79 (11.1%)	45 (57.0%)	1.64	0.99	2.72	
10–25 times	89 (12.5%)	43 (48.3%)	1.16	0.72	1.87	
>25 times	136 (19.1%)	82 (60.3%)	1.88	1.24	2.86	
No. times drew from drug solution in cooker accessed previously by someone else’s used needle (past 6 months)						0.001*
Never	463 (64.9%)	191 (41.3%)	1.00			
1–3 times	107 (15.0%)	60 (56.1%)	1.82	1.19	2.78	
4–9 times	49 (6.9%)	34 (69.4%)	3.23	1.71	6.09	
10–25 times	32 (4.5%)	16 (50.0%)	1.42	0.70	2.92	
>25 times	62 (8.7%)	42 (67.7%)	2.99	1.70	5.25	
No. times drew drug from cooker used by someone else on previous occasion (past 6 months)						<0.001
Never	317 (45.4%)	144 (45.4%)	1.00	-		
1–3 times	90 (12.9%)	28 (31.1%)	0.54	0.33	0.89	
4–9 times	53 (7.6%)	24 (45.3%)	0.99	0.55	1.78	
10–25 times	72 (10.3%)	37 (51.4%)	1.27	0.76	2.12	
>25 times	167 (23.9%)	100 (59.9%)	1.79	1.23	2.62	
No. times drew drugs from cotton previously used by someone else (past 6 months)						0.001*
Never	323 (45.2%)	139 (43.0%)	1.00	-		
1–3 times	107 (15.0%)	42 (39.3%)	0.86	0.55	1.34	
4–9 times	81 (11.3%)	43 (53.1%)	1.50	0.92	2.44	
10–25 times	77 (10.8%)	40 (52.0%)	1.43	0.87	2.36	
>25 times	126 (17.7%)	79 (62.7%)	2.23	1.46	3.40	
No. times drew drugs from cotton accessed previously by someone else with used needle (past 6 months)						0.001*
Never	470 (65.8%)	195 (41.5%)	1.00	-		
1–3 times	78 (10.9%)	36 (46.1%)	1.21	0.75	1.96	
4–9 times	61 (8.5%)	42 (68.9%)	3.12	1.76	5.52	
10–25 times	28 (3.9%)	17 (60.7%)	2.18	1.00	4.76	
>25 times	77 (10.8%)	53 (68.8%)	3.11	1.86	5.22	
No. times using rinse water previously accessed by someone else (past 6 months)						0.02*
Never	346 (48.5%)	154 (44.5%)	1.00	-		
1–3 times	109 (15.3%)	46 (42.2%)	0.91	0.59	1.41	
4–9 times	68 (9.5%)	32 (47.1%)	1.11	0.66	1.87	
10–25 times	57 (8.0%)	31 (54.4%)	1.49	0.85	2.61	
>25 times	134 (18.8%)	80 (59.7%)	1.84	1.23	2.77	
Frequency of cleaning skin with alcohol before injecting (past 6 months)						<0.001*
Never	241 (33.8%)	134 (55.6%)	1.00	-		
occasionally (1–25%)	262 (36.7%)	134 (51.1%)	0.84	0.59	1.19	
about half the time (26–74%)	76 (10.6%)	27 (35.5%)	0.44	0.26	0.75	
most of the time (75–99%)	62 (8.7%)	28 (45.2%)	0.66	0.38	1.15	
Always	73 (10.2%)	20 (27.4%)	0.30	0.17	0.53	
Frequency of cleaning skin with soap and water before injecting (past 6 months)						0.16*
Never	437 (61.2%)	220 (50.3%)	1.00	-		
occasionally (1–25%)	172 (24.1%)	81 (47.1%)	0.88	0.62	1.15	
about half the time (26–74%)	42 (5.9%)	14 (33.3%)	0.49	0.25	0.96	
most of the time (75–99%)	34 (4.8%)	13 (38.2%)	0.61	0.30	1.25	
Always	29 (4.1)	15 (51.7%)	1.06	0.50	2.24	
Frequency of cleaning hands with soap and water before injecting (last 6 months)						0.02*
Never	278 (38.9%)	141 (50.7%)	1.00	-		
occasionally (1–25%)	213 (29.8%)	109 (31.8%)	1.02	0.71	1.46	
about half the time (26–74%)	97 (13.6%)	47 (48.5%)	0.91	0.58	1.45	
most of the time (75–99%)	60 (8.4%)	19 (31.7%)	0.45	0.25	0.81	
Always	66 (9.2%)	27 (40.9%)	0.67	0.39	1.16	

*Mantel-Haenszel chi-square test for trend

In multivariable analysis, six variables representing potential mechanisms or explanations of HCV transmission were independently associated with HCV infection: age, more years since first drug injection, injection frequency, injecting with a needle/syringe previously used by a larger number of people, dividing drug using a syringe used previously with a larger number of people, and drawing drugs from cotton used previously by someone else (Table 5A). In multiple logistic regression models that adjusted for these six variables, social and behavioral characteristics that remained significantly associated with HCV seroprevalence included having injected cocaine or crack, having overdosed, having been arrested for possession of drug residue or paraphernalia, older age of person who first injected participant, less frequent cleaning of skin with alcohol before injection, lacking confidence in being able to avoid HCV infection, and injecting primarily in public or outdoor spaces (Table 5).

**Table 5:** Factors associated with HCV antibody in multivariable analysis*.

Variable	AOR (95% CI)	P
A. Potential explanatory variables		
Age †	2.11 (1.59–2.80)	<0.001
Years since first drug injection †	1.63 (1.36–1.97)	<0.001
Injection frequency (past 6 months) †	1.26 (1.09–1.45)	0.001
No. people who used a needle/syringe before participant (past 6 months)	1.24 (1.07–1.44)	0.004
No. people divided drugs with by drawing into syringe used by someone else before participant (past 6 months)	1.43 (1.11–1.86)	0.006
No. times drew drugs from cotton accessed previously by someone else (past 6 months)	1.20 (1.02–1.40)	0.024
B. Demographic characteristics		
Gender (male)	1.08 (0.75–1.53)	0.686
High School Diploma or GED	0.99 (0.66–1.48)	0.957
Currently employed	0.72 (0.31–1.65)	0.437
Currently homeless	1.17 (0.80–1.72)	0.416
C. Characteristics of first illicit drug injection		
Administered own first injection	0.98 (0.64–1.51)	0.939
Age of person who administered first injection † ‡	2.79 (1.24–6.26)	0.013
Before first injection knew HIV could be transmitted by sharing needles	1.03 (0.54–1.95)	0.932
Before first injection knew hepatitis could be transmitted by sharing needles	0.70 (0.48–1.03)	0.070
Before first injection knew hepatitis could be transmitted by sharing cottons, cookers, or rinse water	0.99 (0.69–1.43)	0.970
D. Past injection practices and other experiences		
Ever injected crack/cocaine	2.10 (1.30–3.42)	0.002
Ever injected pharmaceutical pain killers	1.00 (0.60–1.67)	0.988
Ever injected crystal meth	1.10 (0.76–1.58)	0.615
Ever injected ketamine	0.94 (0.63–1.40)	0.769
Ever overdosed	1.91 (1.34–2.74)	<0.001
Ever arrested solely for drug residue or possession of syringe or needle	3.19 (1.94–5.25)	<0.001
Ever been given money or drugs in exchange for sex?	1.09 (0.75–1.61)	0.644
E. Current injection practices		
Injected most commonly in public/outdoors (past 6 months)	1.91 (1.33–2.75)	<0.001
*Injected most commonly in public/outdoors (past 6 months)* §	*1*.*98 (1*.*34–2*.*92)*§	*0*.*001*§
No. times injected with needle/syringe used previously by someone else(past 6 months)	1.12 (0.93–1.34)	0.240
No. times divided drugs by drawing into syringe used on a previous occasion by someone else (past 6 months)	1.01 (0.80–1.26)	0.956
No. times drew from drug solution in cooker accessed previously by someone else (past 6 months)	0.98 (0.86–1.12)	0.791
No. times drew from drug solution in cooker accessed previously by someone else’s used needle (past 6 months)	1.02 (0.83–1.25)	0.850
No. times drew drug from cooker used by someone else on previous occasion	1.00 (0.98–1.02)	0.980
No. times drawing drugs from cotton accessed previously by someone else with used needle (past 6 months)	0.95 (0.80–1.12)	0.535
No. times using rinse water previously accessed by someone else (past 6 months)	0.99 (0.87–1.14)	0.932
Frequency of cleaning skin with alcohol before injecting (past 6 months)	0.86 (0.74–0.98)	0.029
Frequency of cleaning your skin with soap and water before injecting (past 6 months)	0.98 (0.83–1.16)	0.820
Frequency of cleaning your hands with soap and water before injecting (past 6 months)	0.95 (0.82–1.09)	0.438
F. Self-Perceived risk		
Self-reported HCV-positive	16.94 (9.26–31.01)	<0.001
Confidence in avoiding hepatitis C virus infection (4-point Likert scale) ^||^	1.51 (1.21–1.90)	<0.001

*All values are adjusted for the first 6 variables in the table (Potential explanatory variables). All variables in model are categorical variables unless otherwise noted.

^†^ Log-transformed continuous variable

^‡^ Excluding those who administered their own first injection

^§^ Adjusted for homelessness

^||^Excluded self-reported HCV-positive.

We asked participants about their current injection practices, but prevalent infections may have been acquired in the remote past, and participants who knew they were HCV-positive might have reduced or systematically underreported their current injection practices. To assess whether such bias caused underestimation of the effects of these practices on HCV seropositivity we re-examined these effects after excluding such participants. Doing so did not significantly alter the estimates of these effects (S1 and S2 Tables).

## Discussion

We found HCV antibodies in 48% of the young people participating in our study. The seroprevalence was not significantly associated with calendar time during our study (from 2005 through 2012), and in fact among those age 18–29 was 45%, nearly the same as that in a study of 18-29-year-old PWID on the Lower East Side during 1997–1998 (42%)[24], suggesting that it remained relatively constant for 15 years. The estimated seroprevalence in an older sample of PWID entering treatment for substance use citywide was somewhat higher (67%) and also showed little change during a similar period of time (2000–2013) [15]. Disturbingly, 20.2% of our participants who had injected drugs for less than one year had already been infected. These new initiates to injection drug use are seldom linked to harm reduction services and needle syringe exchanges programs at the time they initiate their injection career[29]. This, coupled with the fact that only two-thirds of participants in our study were aware that hepatitis could be transmitted through sharing of needles and syringes when they first injected drugs, highlights the need for HCV prevention strategies to reach young people when they initiate injecting drugs or before. Drugs and information about drugs travel rapidly through indigenous networks among people who use drugs. Because illicit drug use is an underground activity, capitalizing on these indigenous channels to provide information about HCV prevention and other health interventions (such as overdose prevention) may be the most effective way to reach young people before they initiate injection. Research is needed to explore this potential approach. Community empowerment interventions, for example, have been effective in mobilizing indigenous social networks among PWID and other stigmatized communities[30–32].

Older age, more years since first drug injection, higher injection frequency, and injecting with needle/syringe used by another person were significantly associated with HCV seropositivity. In addition, we were able to identify more granular sharing behaviors and practices within the injection pathway that were independent risk factors for HCV infection, such as drawing drugs from a previously used cotton and splitting drug solution with a previously used syringe. This emphasizes that HCV prevention messages should explain how drugs can get contaminated with HCV at each step of the process of preparing and injecting them. Interestingly there was an independent association between HCV seropositivity and the *number of people* who had used a syringe before the participant, but not the *number of times* a participant used a previously used syringe. This probably reflects the high likelihood of infection after using a syringe, even once, that was previously used by someone with the virus, so that the frequency of exposure is less important than the likelihood of encountering an HCV-infected person. But it also suggests that reducing the number of their partners may enable PWID to reduce their risk of infection and reduce disease transmission in their community.

What remains a challenge in the prevention of blood-borne pathogen transmission among PWID is ensuring the uptake of harm reduction services, a consistent supply of clean injection equipment, and interventions to reach those initiating injection drug use to help them avoid high-risk injection. Despite access to harm reduction services, over half of our study participants reported injecting drugs with previously used syringes during the previous six months, and more than half reported injecting drugs that had been divided with a previously used needle/syringe.

Although HIV remains a major risk for PWID, the prevalence and incidence of HIV among people who use drugs, including those in our study, remain significantly less than the prevalence and incidence of HCV. However, 612 (86%) of our 714 participants reported having prior HIV testing; in comparison only 466 (65%) participants had previously been tested for HCV. These data suggest that HIV prevention measures have permeated the drug-using community, but HCV prevention is lagging. The high prevalence of HCV infection, high and increasing mortality associated with HCV infection, recent advances in HCV treatment, and potential role of HCV treatment of PWID in limiting transmission (treatment-as-prevention), provide compelling reasons for policy makers to make public health investments in HCV prevention, screening, and treatment.

Over half of our study participants injected most frequently in public or outdoor locations, which we found to be independently associated with HCV antibody positivity. To our knowledge this is the first study to demonstrate this association, although prior studies have demonstrated the association of shooting galleries use [33,34] and homelessness[35] with HCV seropositivity. Public and outdoor injection drug use may often be more rushed than home-based injection because of the dangers of being observed or arrested, and the rushed nature of this practice may make the implementation of safe injection practices more difficult. Tools for injection hygiene such as running water and sterile injection supplies are also likely to be less available in public and outdoor locations. Over the last several years there has been growing interest in and support for supervised injection facilities, to reduce the sequelae of unsafe drug injection. Ithaca, New York is the first municipality in the United States to announce plans to create such a facility[36], and now Seattle as well. Studies have demonstrated that these facilities significantly reduce overdose mortality, public injection, and publically discarded needles in the area surrounding the site[37,38]. Our study, showing a strong and persistent association between public/outdoor injection and HCV infection, suggests that providing PWID access to supervised indoor injection facilities may also reduce HCV transmission. Further studies are needed to evaluate this potential and better inform public policy discussion of this intervention.

Other findings suggest additional possible avenues for effective responses to the HCV epidemic. First, two-thirds of our participants were homeless, and their HCV prevalence was higher than that of the other participants. Our multivariable analysis suggests that their higher risk may have been explained by risky injection practices, but those practices may have resulted from their homelessness. Safe, quality housing has been shown to improve a number of health outcomes. It may also decrease the spread of HCV. Second, drug overdose was a common life-threatening event in our population, and the 57% of our study participants who had overdosed had more than twice the likelihood of HCV infection. This suggests that drug overdose rates not only signal the need for overdose prevention interventions, but are also likely a valuable sentinel marker for communities where HCV transmission, a silent and less visible consequence of drug injection, is occurring—identifying the need for intervention to prevent HCV infection. Third, participants’ assessments of their own risk added significantly to the predictive value of our model, indicating that they are often aware of how they incur risk beyond our ability to ascertain it through careful questioning. This suggests the importance of involving people who inject drugs in designing programs to protect them so they can benefit from their insight and target sources of risk appropriately. Fourth, New York State Law permits possession of hypodermic needles and syringes obtained from an authorized syringe exchange program, but 19% of our participants reported having been arrested solely for possessing a syringe or the drug residue in it, and they had a nearly 5-fold increased prevalence of HCV. This finding underscores the difficulty of maintaining an adequate supply of sterile injection equipment, and focusing on healthier behaviors such as safer injection practices, when one is simultaneously trying to avoid arrest. Arresting people for carrying syringes directly contravenes the benefit of public health efforts to assure access to sterile syringes. Collaboration between communities, public health agencies, and law enforcement so that public health and public safety efforts are coordinated and cooperative, could reduce problems caused when these entities work at cross-purposes[39,40].

This study has several limitations. First, the ability to generalize this study to all injection drug users is limited by the fact that some of the recruitment for the study occurred at a needle and syringe program. Whether the injection practices of drug users seeking out harm reduction services is significantly different from those not engaged with these services could not be explored in this study.

Second, the study relied on self-report, which is known to suffer from imperfect recall and socially desirable reporting. Additionally, participants previously diagnosed with HCV may have made greater efforts to identify preceding potential transmission events than those without a prior diagnosis (recall bias). Third, causality cannot be inferred from associations between demographics or behavior and HCV seropositivity (especially in a cross-sectional study).

Finally, this study took place in New York City where independent, community-based, syringe services have existed for more than two decades. Thus our findings cannot be generalized to different areas of the country, especially non-urban settings, where injection drug use is increasing, access to clean injection equipment and harm reduction services is limited[17], and the drugs injected may be different. Still, many of our findings, such as the risks of dividing a dose of drugs with used syringes, the number of different persons with whom one shares syringes, and the length of time injecting, are likely to be applicable to other areas.

In summary, our findings underscore the high rates of HCV infection in young people injecting drugs in New York, with a significant proportion (20%) being infected within their first year of injection. These early seroconversions highlight the need to refine current harm reduction interventions to enhance their ability to reach new drug injectors to further impact the transmission of HCV through injection drug use. There remains a need to reach young people initiating drug injection, perhaps through community mobilization interventions, and provide harm reduction messages, supplies, and HCV testing to enable them to avoid high-risk injection practices and HCV infection. These messages must convey the risks of less apparent high-risk practices such as dividing drugs with a used syringe, and emphasize the benefits of reducing the number of people with whom one has blood contact if such contact can’t be eliminated entirely. Overdose rates should serve as a sentinel to identify the need for HCV prevention intervention. Law enforcement should be engaged as partners in advancing public health and public safety. Finally, our study highlights the potential role for supervised injection facilities to reduce public and outdoor injection and decrease the transmission of HCV.

## Supporting information

S1 Table HCV seroprevalence by recent injection practices, young people who inject drugs, New York City, 2005–2012 (Excluding 180 participants who self-reported positive HCV status). (DOCX)

S2 Table Current injection practices associated with HCV antibody in multivariate analysis* (Excluding 180 participants who self-reported positive HCV status). (DOCX)

S3 Table HCV seroprevalence by age and years since first drug injection. (DOCX)
