# Supplementary material for: Risk factors for hepatitis C seropositivity among young people who inject drugs in New York City: Implications for prevention
Source: PLoS One. 2017 May 19;12(5):e0177341. doi: 10.1371/journal.pone.0177341 (PMC5438142; doi:10.1371/journal.pone.0177341)
Supplement: S1 Table — (DOCX) [file pone.0177341.s001.docx]

| Variable | Overall | HCV Ab (+) | Unadjusted OR | | | 95% CI | | p |
| --- | --- | --- | --- | --- | --- | --- | --- | --- |
| **TOTAL** | 534 (100%) | 178 (33.3%) |  | | |  |  |  |
| Injection frequency (injections/month) | | | | | | | | <0.001* |
| <30 | 165 (30.9%) | 37 (22.4%) | 1 | | - | | |  |
| 30-59 | 84 (15.7%) | 21 (25.0%) | 1.15 | | 0.62 | | 2.13 |  |
| 60-89 | 90 (16.9%) | 37 (41.1%) | 2.42 | | 1.38 | | 4.21 |  |
| 90-149 | 109 (20.4%) | 48 (44.0%) | 2.72 | | 1.61 | | 4.61 |  |
| 150-299 | 65 (12.2%) | 23 (35.4%) | 1.89 | | 1.01 | | 3.54 |  |
| ≥300 | 21 (3.9%) | 12 (57.1%) | 4.61 | | 1.8 | | 11.79 |  |
| Location where injected drugs most (last 6 months) | | | | | | | | 0.11 |
| Your or primary partner's home | 150 (28.1%) | 41 (27.3%) | 1 | |  | |  |  |
| Home of a friend or relative | 58 (10.9%) | 14 (24.1%) | 0.85 | | 0.42 | | 1.7 |  |
| Public or outdoor space | 279 (52.3%) | 106 (38.0%) | 1.63 | | 1.06 | | 2.51 |  |
| Other indoor space | 28 (5.2%) | 10 (35.7%) | 1.48 | | 0.63 | | 3.46 |  |
| Other | 19 (3.6%) | 7 (36.8%) | 1.55 | | 0.57 | | 4.21 |  |
| No. times injected with needle/syringe used previously by someone else(past 6 months) | | | | | | | | 0.001* |
| Never | 247 (46.3%) | 63 (25.5%) | 1 | | - | | |  |
| 1-3 times | 110 (20.6%) | 37 (33.6%) | 1.48 | | 0.91 | | 2.41 |  |
| 4-9 times | 56 (10.5%) | 20 (35.7%) | 1.62 | | 0.88 | | 3.01 |  |
| 10-25 times | 45 (8.4%) | 23 (51.1%) | 3.05 | | 1.59 | | 5.85 |  |
| >25 times | 75 (14.1%) | 35 (46.7%) | 2.56 | | 1.49 | | 4.37 |  |
| No. people who used a needle/syringe before participant (past 6 months) | | | | | | | | 0.002* |
| None | 252 (48.7%) | 65 (25.8%) | 1 | | - | | |  |
| 1 person | 130 (25.2%) | 49 (39.7%) | 1.74 | | 1.11 | | 2.74 |  |
| 2 people | 50 (9.7%) | 17 (34.0%) | 1.48 | | 0.77 | | 2.84 |  |
| 3 people | 28 (5.4%) | 14 (50.0%) | 2.88 | | 1.3 | | 6.36 |  |
| 4-9 people | 43 (8.3%) | 20 (46.5%) | 2.5 | | 1.29 | | 4.85 |  |
| 10-25 people | 12 (2.3%) | 8 (66.7%) | 5.75 | | 1.68 | | 19.74 |  |
| >25 people | 2 (0.4%) | 1 (50.0%) | 2.88 | | 0.18 | | 46.66 |  |
| No. times divided drugs by drawing into syringe used on a previous occasion by someone else (past 6 months) | | | | | | | | <0.001* |
| Never | 383 (71.7%) | 105 (27.4%) | 1 | | - | | |  |
| 1-3 times | 47 (8.8%) | 14 (29.8%) | 1.12 | | 0.58 | | 2.18 |  |
| 4-9 times | 29 (5.4%) | 15 (51.7%) | 2.84 | | 1.32 | | 6.08 |  |
| 10-25 times | 39 (7.3%) | 22 (56.4%) | 3.43 | | 1.75 | | 6.71 |  |
| >25 times | 36 (6.7%) | 22 (61.1%) | 4.16 | | 2.05 | | 8.43 |  |
| No. people divided drugs with by drawing into syringe used by someone else before participant (past 6 months) | | | | | | | | <0.001* |
| None | 373 (72.3%) | 105 (28.2%) | 1 | | - | | |  |
| 1 person | 102 (19.8%) | 40 (39.2%) | 1.65 | | 1.04 | | 2.6 |  |
| 2 people | 20 (3.9%) | 13 (65.0%) | 4.74 | | 1.84 | | 12.21 |  |
| 3 people | 6 (1.2%) | 3 (50.0%) | 2.55 | | 0.51 | | 12.85 |  |
| 4-9 people | 13 (2.5%) | 10 (76.9%) | 8.51 | | 2.3 | | 31.53 |  |
| 10-25 people | 2 (0.4%) | 2 (100%) | - | | - | | - |  |
| >25 people | 0 (0.0%) | - | - | | - | | - |  |
| No. times drew from drug solution in cooker accessed previously by someone else (past 6 months) | | | | | | | | 0.001* |
| Never | 207 (38.8%) | 63 (30.4%) | 1 | | - | | |  |
| 1-3 times | 106 (19.6%) | 24 (22.6%) | 0.67 | | 0.39 | | 1.15 |  |
| 4-9 times | 60 (11.2%) | 29 (48.3%) | 2.13 | | 1.19 | | 3.84 |  |
| 10-25 times | 62 (11.6%) | 17 (27.4%) | 0.86 | | 0.46 | | 1.62 |  |
| >25 times | 99 (18.5%) | 45 (45.5%) | 1.9 | | 1.16 | | 3.12 |  |
| No. times drew from drug solution in cooker accessed previously by someone else’s used needle (past 6 months) | | | | | | | | <0.001* |
| Never | 362 (67.9%) | 101 (27.9%) | 1 | | - | | |  |
| 1-3 times | 76 (14.3%) | 31 (40.8%) | 1.78 | | 1.07 | | 2.97 |  |
| 4-9 times | 34 (6.4%) | 21 (61.8%) | 4.17 | | 2.01 | | 8.65 |  |
| 10-25 times | 24 (4.5%) | 8 (33.3%) | 1.29 | | 0.54 | | 3.11 |  |
| >25 times | 37 (6.9%) | 17 (46.0%) | 2.2 | | 1.11 | | 4.36 |  |
| No. times drew drug from cooker used by someone else on previous occasion | | | | | | | |  |
| Never | 233 (44.1%) | 68 (29.2%) | 1 | | - | | | 0.002 |
| 1-3 times | 78 (14.7%) | 17 (21.8%) | 0.68 | | 0.37 | | 1.24 |  |
| 4-9 times | 41 (7.8%) | 13 (31.7%) | 1.13 | | 0.55 | | 2.30 |  |
| 10-25 times | 54 (10.2%) | 21 (38.9%) | 1.54 | | 0.83 | | 2.86 |  |
| >25 times | 123 (23.3%) | 57 (46.3%) | 2.10 | | 1.33 | | 3.30 |  |
| No. times drew drugs from cotton previously used by someone else (past 6 months) | | | | | | | | 0.011* |
| Never | 244 (45.7%) | 71 (29.1%) | 1 | | - | | |  |
| 1-3 times | 86 (16.1%) | 23 (26.7%) | 0.89 | | 0.51 | | 1.54 |  |
| 4-9 times | 55 (10.3%) | 19 (34.6%) | 1.29 | | 0.69 | | 2.39 |  |
| 10-25 times | 58 (10.9%) | 21 (36.2%) | 1.38 | | 0.76 | | 2.52 |  |
| >25 times | 91 (17.0%) | 44 (48.4%) | 2.28 | | 1.39 | | 3.74 |  |
| No. times drew drugs from cotton accessed previously by someone else with used needle (past 6 months) | | | | | | | | <0.001* |
| Never | 360 (67.4%) | 97 (26.9%) | 1 | | - | | |  |
| 1-3 times | 64 (12.0%) | 24 (37.5%) | 1.63 | | 0.93 | | 2.84 |  |
| 4-9 times | 38 (7.1%) | 20 (52.6%) | 3.01 | | 1.53 | | 5.93 |  |
| 10-25 times | 20 (3.8%) | 9 (5.1%) | 2.22 | | 0.89 | | 5.52 |  |
| >25 times | 52 (9.7%) | 28 (15.7%) | 3.16 | | 1.75 | | 5.72 |  |
| No. times using rinse water previously accessed by someone else (past 6 months) | | | | | | | | 0.015* |
| Never | 263 (49.3%) | 80 (30.4%) | 1 | | - | | |  |
| 1-3 times | 79 (14.8%) | 19 (24.1%) | 0.72 | | 0.41 | | 1.29 |  |
| 4-9 times | 51 (9.6%) | 16 (31.4%) | 1.05 | | 0.55 | | 2 |  |
| 10-25 times | 43 (8.0%) | 19 (44.2%) | 1.77 | | 0.94 | | 3.49 |  |
| >25 times | 98 (18.4%) | 44 (44.9%) | 1.86 | | 1.16 | | 3 |  |
| Frequency of cleaning skin with alcohol before injecting (last 6 months) | | | | | | | | 0.001* |
| Never | 169 (31.7%) | 66 (39.1%) | 1 | | - | | |  |
| occasionally (1-25%) | 196 (36.7%) | 73 (37.2%) | 0.93 | | 0.61 | | 1.41 |  |
| about half the time (26-74%) | 60 (11.2%) | 11 (18.3%) | 0.35 | | 0.17 | | 0.72 |  |
| most of the time (75-99%) | 49 (9.2%) | 18 (36.7%) | 0.91 | | 0.47 | | 1.75 |  |
| Always | 60 (11.2%) | 10 (16.7%) | 0.31 | | 0.15 | | 0.66 |  |
| Frequency of cleaning your skin with soap and water before injecting (past 6 months) | | | | | | | | 0.192* |
| Never | 329 (61.6%) | 119 (36.2%) | 1 | | - | | |  |
| occasionally (1-25%) | 125 (23.4%) | 36 (28.8%) | 0.71 | | 0.46 | | 1.12 |  |
| about half the time (26-74%) | 32 (6.0%) | 5 (15.6%) | 0.33 | | 0.12 | | 0.87 |  |
| most of the time (75-99%) | 26 (4.9%) | 9 (34.6%) | 0.93 | | 0.4 | | 2.16 |  |
| Always | 22 (4.1) | 9 (40.9%) | 1.22 | | 0.51 | | 2.94 |  |
| Frequency of cleaning your hands with soap and water before injecting (past 6 months) | | | | | | | | 0.223* |
| Never | 205 (38.4%) | 71 (34.6%) | | 1 | - | | |  |
| occasionally (1-25%) | 156 (29.2%) | 56 (35.9%) | | 1.06 | 0.68 | | 1.63 |  |
| about half the time (26-74%) | 72 (13.5%) | 26 (36.1%) | | 1.07 | 0.61 | | 1.87 |  |
| most of the time (75-99%) | 48 (9.0%) | 9 (18.8%) | | 0.44 | 0.2 | | 0.95 |  |
| Always | 53 (9.9%) | 16 (30.2%) | | 0.54 | 0.42 | | 1.57 |  |

*****Mantel-Haenszel chi-square test for trend
