# Supplementary material for: Risk factors for hepatitis C seropositivity among young people who inject drugs in New York City: Implications for prevention
Source: PLoS One. 2017 May 19;12(5):e0177341. doi: 10.1371/journal.pone.0177341 (PMC5438142; doi:10.1371/journal.pone.0177341)
Supplement: S2 Table — (DOCX) [file pone.0177341.s002.docx]

| Variable | AOR (95% CI) | P |
| --- | --- | --- |
| A. Potential explanatory variables |  |  |
| Age † (per year) | 2.18 (1.58-3.01) | <0.001 |
| Years since first drug injection † | 1.30 (1.07-1.59) | 0.01 |
| Injection frequency (past 6 months) † | 1.30 (1.10-1.55) | 0.003 |
| No. people who used a needle/syringe before participant (past 6 months) | 1.20 (1.01-1.43) | 0.035 |
| No. people divided drugs with by drawing into syringe used by someone else before participant (past 6 months) | 1.54 (1.16-2.05) | 0.003 |
| No. times drew drugs from cotton accessed previously by someone else (past 6 months) | 1.17 (0.96-1.39) | 0.091 |
| B. Current injection practices |  |  |
| Injected most commonly in public/outdoors (past 6 months) | 1.86 (1.21-2.86) | 0.004 |
| No. times injected with needle/syringe used previously by someone else(past 6 months) | 1.05 (0.84-1.31) | 0.691 |
| No. times divided drugs by drawing into syringe used on a previous occasion by someone else (past 6 months) | 1.09 (0.86-1.39) | 1.39 |
| No. times drew from drug solution in cooker accessed previously by someone else (past 6 months) | 0.96 (0.82-1.13) | 0.613 |
| No. times drew from drug solution in cooker accessed previously by someone else’s used needle (past 6 months) | 0.91 (0.72-1.16) | 0.443 |
| No. times drew drug from cooker used by someone else on previous occasion | 1.00 (0.98-1.03) | 0.785 |
| No. times drew drugs from cotton accessed previously by someone else with used needle (past 6 months) | 0.91 (0.74-1.12) | 0.376 |
| No. times using rinse water previously accessed by someone else (past 6 months) | 0.96 (0.82-1.13) | 0.65 |
| Frequency of cleaning skin with alcohol before injecting (past 6 months) | 0.87 (0.74-1.03) | 0.105 |
| Frequency of cleaning your skin with soap and water before injecting (past 6 months) | 0.99 (0.82-1.21) | 0.957 |
| Frequency of cleaning your hands with soap and water before injecting (past 6 months) | 0.99 (0.84-1.16) | 0.871 |

| *All values are adjusted for the first 6 variables (hypothesized explanatory variables) | |
| --- | --- |
| † Log-transformed |  |
