# Supplementary material for: Risk factors for hepatitis C seropositivity among young people who inject drugs in New York City: Implications for prevention
Source: PLoS One. 2017 May 19;12(5):e0177341. doi: 10.1371/journal.pone.0177341 (PMC5438142; doi:10.1371/journal.pone.0177341)
Supplement: S3 Table — (DOCX) [file pone.0177341.s003.docx]

|  | No. (%) of participants | No. (%) HCV Ab (+) | No. (%) of participants | No. (%) HCV Ab (+) |
| --- | --- | --- | --- | --- |
|  |  |  |  |  |
|  | Injected Drugs ≤5 years | | Injected drugs >5 years | |
| TOTAL | 372 (52.1%) | 118 (31.7%) | 342 (47.9) | 251 (62.4%) |
| Age, y |  |  |  |  |
| 18-19 | 91 | 17 (18.7%) | 8 | 4 (50.0%) |
| 20-24 | 13 | 54 (29.5%) | 96 | 62 (64.6%) |
| 25-29 | 63 | 30 (47.6%) | 144 | 95 (66.0%) |
| 30-35 | 22 | 12 (54.5%) | 94 | 64 (68.1%) |
| >35 [36-55] | 13 | 5 (38.5%) | 0 | - |
